# p27kip1 at the crossroad between actin and microtubule dynamics

**DOI:** 10.1186/s13008-019-0045-9

**Published:** 2019-04-01

**Authors:** Gian Luca Rampioni Vinciguerra, Francesca Citron, Ilenia Segatto, Barbara Belletti, Andrea Vecchione, Gustavo Baldassarre

**Affiliations:** 1 Division of Molecular Oncology, Centro di Riferimento Oncologico di Aviano (CRO), IRCCS, National Cancer Institute, 33081 Aviano, Italy; 2 Faculty of Medicine and Psychology, Department of Clinical and Molecular Medicine, University of Rome “Sapienza”, Santo Andrea Hospital, 00189 Rome, Italy

**Keywords:** p27, Microtubule, Actin, Cytoskeleton, Stathmin, Migration, Cancer

## Abstract

The p27^kip1^ protein, mainly known as a negative regulator of cell proliferation, has also been involved in the control of other cellular processes, including the regulation of cytoskeleton dynamics. Notably, these two functions involve distinct protein domains, residing in the N- and C-terminal halves, respectively. In the last two decades, p27^kip1^ has been reported to interact with microtubule and acto-myosin cytoskeletons, both in direct and indirect ways, overall drawing a picture in which several factors play their role either in synergy or in contrast one with another. As a result, the role of p27^kip1^ in cytoskeleton dynamics has been implicated in cell migration, both in physiologic and in neoplastic contexts, modulating cytokinesis, lipid raft trafficking, and neuronal development. Recently, two distinct papers have further reported a central role for p27^kip1^ in the control of microtubule stability and post-translational modifications, dissecting the interaction between p27^kip1^ and α-tubulin-acetyl-transferase (α-TAT), an enzyme involved in the stability of microtubules, and protein-regulator of cytokinesis 1 (PRC1), a nuclear regulator of the central spindle during mitosis. In light of these recent evidences, we will comment on the role of p27^kip1^ on cytoskeleton regulation and its implication for cancer progression.

## Background

p27^kip1^ (hereafter p27) is a member of the CIP/KIP family of cyclin-dependent kinase (CDK) inhibitors (CKI), also comprising p21^waf1^ and p57^kip2^. These CKIs are negative regulators of cell proliferation, mainly working by impairing the activity of cyclin-CDK complexes in the cell nucleus [1].

However, their involvement in CDK-independent functions, such as cell migration and, more in general, cytoskeletal remodeling have also emerged [1]. Indeed, all three members of the CIP/KIP CKI family have been shown to interfere with the pathway of RhoA, a GTPase that regulates actin remodeling and cell migration when located in the cytoplasm [1]. In particular, it has been reported that cytoplasmic p21^waf1^ can directly bind Rho-kinase (ROCK), inhibiting stress fiber formation and regulating neurite outgrowth in Ras-transformed NIH3T3 cells [2, 3], and that p57^kip2^ can reduce motility in HeLa cells, regulating the actin cytoskeleton via LIM-kinase1, a well-known downstream effector of ROCK [4].

The capability to shuttle from the nucleus to the cytoplasm and viceversa, according to the cell cycle phase, is at the basis of these CKI functions [1]. With regard to p27, its subcellular localization is mediated by two different domains, namely the Nuclear Export Signal (NES) and the Nuclear Localization Signal (NLS), and it is strictly regulated by phosphorylation of key residues by several kinases, such as AKT, ERK1/2 and RSK1/2. Then, phosphorylation on tyrosine residues within the CDK binding domain modulates the CDK inhibitor activity [1].

The role of p27 in cytoskeleton dynamics is less consolidated and has long presented controversial aspects. In 1998, the group of Dowdy et al. firstly reported that hepatocellular carcinoma cells, transduced with TAT-p27 fusion protein, rearranged actin cytoskeleton forming lamellipodia and filopodia in a 2D-assay [5]. Later on, the same authors postulated that this pro-migratory effect of p27 required Rac1, a GTPase protein known as a regulator of actin remodeling [6]. Many other evidences from different groups contributed to clarify the role(s) of p27 in the control of cytoskeletal dynamics, in some cases involving Rac1, RhoA or citron-kinase and resulting in the control of actin assembly and actomyosin contractile ring formation during cytokinesis [7–9]; in other cases involving stathmin 1 (hereafter referred as stathmin, STM) and resulting in the control of microtubule (MT) dynamics [10]. As expected, p27 regulation of cytoskeleton organization affected cell shape and motility and, depending on the context, resulted in either pro- or anti-migratory effects, as recently reviewed by Sharma et al [11].

Here, we will briefly review and comment the new insights coming from most recently published data, supporting that MT-regulation by p27 plays a pivotal role in several cellular processes that range from mitotic division and neuronal differentiation to cell motility, invasion and metastasis control.

## Main text

### p27 modulates microtubule acetylation and stability via α-TAT1 and PRC1

Nguyen et al. have recently observed that, in murine cortical neurons, p27 is located in multiple cytoplasmic compartments, including both dendrites and axons [12]. Projections from p27 knock out (p27^KO^) neurons displayed a reduced instantaneous and average transport velocity compared to wild-type (p27^WT^) controls. Moreover, p27^KO^ neurons were also characterized by a reduction in acetylation of α-tubulin and the inhibition of Histone-deacetylase-6 (HDAC6) completely rescued neuronal transport velocity.

Looking at α-tubulin-acetyltransferase-1 (α-TAT1), the main enzyme involved in α-tubulin acetylation, the authors described a decrease at protein- but not at mRNA-level. Thus, a reduced MT-acetylation in p27^KO^ neurons could be due to a reduction in α-TAT1 half-life time. In support of that, treatment with proteasome inhibitors restored axonal trafficking. Looking for the molecular pathway(s) that could mediate this phenotype, they co-immunoprecipitated p27 with α-TAT1 and discovered that this interaction took place *via* its C-terminal portion (89–198 amino acids, aa) and could positively modulate α-TAT1 stability, at least in HEK293 cells.

Altogether, the authors demonstrated that the C-terminal domain of p27 directly interacts with α-TAT1, thus preventing its proteasome-mediated degradation and modulating MT-acetylation and axonal transport. Overall, these results strongly supported that p27 impacts on MT cytoskeleton dynamics in neurons.

Dissecting the role of p27 in this field, new insights have also been provided by the recent work of Besson et al. who reported for the first time the interaction of p27 with protein regulator of cytokinesis 1 (PRC1) [13].

PRC1 is an MT-associated protein, mainly localized in the nucleus, that plays a critical role in mitotic central spindle formation by crosslinking antiparallel MTs. PRC1 expression is increased in several types of cancer and correlates with poor prognosis, possibly by promoting multi-nucleation and aneuploidy, which are well-known causes of chromosome instability.

PRC1 was identified in a protein array probed with recombinant human p27 and the authors then confirmed the direct binding between PRC1 and p27 by co-immunoprecipitation assay in HEK293 cells. In particular, this interaction was not detected with a C-terminal truncated variant of p27 (p27^1–190^), indicating that the last eight aa were necessary to bind PRC1. In vitro, PRC1 was required to form large branched networks of MTs but, in presence of p27, MT bundles were less numerous, smaller and less branched. In vivo, PRC1 overexpressing HeLa cells showed different MT arrangements if co-transfected with either p27^CK−^ (unable to bind cyclin-CDKs) or p27^1–190^. Interestingly, p27^CK−^ (but not p27^1–190^) was effective to prevent the formation of perinuclear thick bundled MTs, typically observed after PRC1 overexpression, indicating that the ability of p27 to impair the hyper-bundling PRC1-dependent phenotype was independent from CDK binding.

Therefore, p27 was able to efficiently counteract the cytokinesis defect and multi-nucleation due to a hyper-expression of PRC1, thus directly linking p27 expression to the control of cell transformation.

### p27 at the crossroad between actin and microtubule dynamics

Interestingly, while p27 N-terminus has been unequivocally recognized as a cyclin/CDK regulator, the C-terminus has been very often linked to the binding and control of proteins that, directly or indirectly, modulate either the actin or the MT cytoskeletons, such as Rac1 [6], RhoA [7], stathmin [10], citron kinase [9] and, in some cases, MTs themselves [8]. In line with these observations, the new data mentioned above demonstrate that also α-TAT1 and PRC1 bind the p27 C-terminus, further supporting its role in controlling the MT-cytoskeleton dynamics and then biological processes such as cytokinesis, cell motility and metastasis formation.

One consideration that clearly emerges from these findings is that many effects observed in cellular assays using p27 deletion or point mutants could be more complex than initially imagined. The observed outcome, in terms of phenotype reported, likely represented the overlap of different activities, the result of peculiar subcellular localizations and differing protein stabilities of the different mutants, as well as, of course, the result of the model system adopted.

For instance, the p27 scatter domain, originally identified by Dowdy et al. in hepatocellular carcinoma cells treated with HGF and transduced with TAT-p27 fusion proteins, was mapped between aa 118–158 and indirectly determined the regulation of Rac1 [6]. Interestingly, it partially overlaps with the RhoA binding mapped by Roberts et al. between aa 86 and 198, using HEK293 cells [7].

Recently, Kriwacki et al. performing titrimetry using isotopically labeled p27 and NMR spectroscopy, identified p27 residues 55–95 as the domain that bound to RhoA, pointing out that this direct interaction had low affinity [14]. In order to reconcile this new finding with previous literature data and experimental evidence demonstrating how isolated p27 N- and C-terminus are not able to recapitulate the RhoA inhibition induced by the full-length p27, the authors hypothesized that p27-RhoA interaction could involve an additional mediator [14].

Remarkably, both the regulation of Rac1 and the binding to RhoA by p27 have been linked to increased cell motility in 2D-assays, *e.g.* HGF-induced scattering or wound healing on plastic culture dishes, respectively for Rac1 and RhoA. The pro-migratory function of p27 has then stimulated the study of its possible role in the control of cancer cell motility and metastasis formation. In 2006, Wu et al generated a p27 mutant lacking the Nuclear Localization Signal (ΔNLS), that was specifically retained in the cytoplasm [15]. In MCF7 breast carcinoma cells this mutant stimulated transwell motility and wound closure in 2D-assays, likely through the regulation of RhoA activity. These data were then further reinforced by the observation that Ras/MEK/MAPK and PI3K/PDK1 pathways drove p27 phosphorylation, resulting in increased RhoA-p27 binding and cell motility [16].

On the contrary, using human sarcoma cells and oncogene-transformed Murine Embryonic Fibroblasts (MEFs) as model systems and 3D-assays to test cell motility, our group observed a strong anti-migratory and anti-metastatic activity of p27, linked to its C-terminal portion (aa 170–198) [10, 17–19]. Furthermore, in xenograft models, the expression of p27^WT^, but not of p27 deleted of the last 28 aa (p27^1–170^), strongly prevented *v*-Src or H-Ras transformed cells from invading blood vessels and forming distant metastasis [17, 19]. The molecular dissection of these anti-migratory effects led to the discovery that the binding of p27 to stathmin via its C-terminus could be regulated by the phosphorylation on T198 and resulted in impaired stathmin activity [10, 20]. Stathmin is an MT-destabilizing protein often overexpressed in metastatic tumors [21]. By interacting with p27, stathmin ability to bind MTs decreased, thereby decreasing its MT-destabilizing activity. Conversely, high stathmin expression/activity favored the acquisition of amoeboid-like cell motility [22], by regulating RhoA in a MT-dependent manner [23]. Accordingly, the amoeboid-like motility that characterized *v*-Src-transformed p27^KO^ or mutant p27^1–170^ fibroblasts could be reverted by RhoA pathway inhibition or by low doses of Taxol, which led to MT-stabilization.

Overall, the scenario emerging from more than 15 years of research on this topic, definitely assigns a role to p27 in the control of cell motility and metastasis through the regulation of MT-dynamics, which is independent from its ability to regulate cyclin/CDK complexes and is linked to its cytoplasmic localization. Yet, in our opinion, a definitive portrait of the role(s) of p27 C-terminus in cancer is still a work-in-progress and presents some unclear aspects that will need to be addressed.

### p27 in neuronal differentiation

The role of p27 in neuronal differentiation (via neurogenin binding) and migration (via RhoA inhibition) is even more complex [24]. We and others originally reported that p27 is necessary for the neuronal differentiation of embryonal carcinoma cells induced by retinoic acid and that p27 ortholog in Xenopus, Xic1, can induce the differentiation of Müller glia from retinoblasts, independently from its ability to block cell cycle progression [25, 26]. Accordingly, it was reported that reduced p27 protein stability, induced by CDK5 inhibition, decreases neuron migration in vivo by cytoskeletal remodeling, in post mitotic human neurons [27]. Despite several insights coming from these studies, the physiological relevance of p27 in neurogenesis has not been fully elucidated. p27^KO^ mice show retinal dysplasia and specific alterations of the neocortex, such as altered number and distribution of the non-GABAergic projection neurons, enlarged cortical thickness as a consequence of increased production of layer II–IV projection neurons [28, 29]. However, these features have been mostly linked to perturbed cell cycle exit in neural progenitor cells, an essential role played by p27 and well elucidated in the in vivo model developed by Roussel et al. [30]. According to their work, mice knocked out for both p19^INK4D^, a member of CDK4-inhibitors family, and p27^KIP1^ exacerbated their inability to keep mature neurons out from the cell-cycle. Consequently, p19/p27 double KO mice showed an abnormal mitotic activity in post-migratory neurons, resulting in a phenotype characterized by bradykinesia, proprioceptive abnormalities, muscle weakness, seizure and death occurring after few weeks from birth, probably because of their inability to feed themselves [30]. More recently, Kempermann et al. reported that mice employed in behavioral activities displayed an upregulation of cytoplasmic p27 in the neurogenic zone of the hippocampus, which is devoted to integrating established memory with new pieces of information, suggesting that p27 could also play a role in post-mitotic neurons [31]. Indeed, it is to note that p27^KO^ mice display a “neurogenesis phenotype” characterized by impaired spatial learning ability if challenged with specific trials requiring hippocampus-dependent strategies [31]. Although this phenotype seems to be linked to a cytoplasmic localization of p27, it has not been clearly linked to alterations of the cytoskeleton dynamics. However, this phenotype is highly reminiscent of the motor behaviour of larvs knocked down for Dacapo (the drosophila ortholog of p27) that showed reduced crawling speed and peristaltic waves followed by climbing defects in adult life [12].

More recently, Nguyen et al. deepened this investigation and reported that p27 controls nucleokinesis and neurite branching by regulating both actin and microtubule cytoskeletons [8]. Now, the new reports by the groups of Nguyen and Besson further increase this complexity, ascribing to α-TAT1 and PRC1, two MT-stabilizing proteins, a role in neurite differentiation, axonal transport and cytokinesis, respectively. Interestingly, the binding of α-TAT1 to p27 is located in the region previously reported to bind RhoA and stathmin and both these proteins have been previously linked to the regulation of neuron migration and axonal transport. Similarly, the binding of PRC1 is located in a p27 region already identified as the one involved in the binding with citron kinase (aa 190–198), also able to regulate cytokinesis.

### p27 in cancer: the unconventional case of an haploinsufficient and intrinsically disordered tumor suppressor

p27 has been always described as an atypical tumor suppressor, for which the concept of haplo-insufficiency has been first defined [29]. In line with this knowledge, from the thorough study of CDKN1B mutation in human cancer we can readily appreciate that p27 is infrequently affected by inactivating mutations or loss of heterozygosity, which are events commonly affecting other tumor suppressors, such as p16^ink4^ and RB. Although rare, frameshift and nonsense mutations are largely predominant and often result in the truncation of p27 protein, leading to loss of the C-terminal domain [32]. Mutations in p27 gene, CDKN1B, have been reported for luminal breast cancer, prostate adenocarcinoma and, particularly frequent, small intestine neuroendocrine tumors and, again, the C-terminal domain is the most frequently mutated region [32]. Since both the MT-interacting domain(s) and the NLS fall in p27 C-terminus, these data strongly suggest that truncation/mutation of p27 protein in this region could fulfill at the same time two critical requirements for cell transformation: it may prevent MT-stabilization inducing, among others, a pro-migratory effect; and it may ensure, by promoting its cytoplasmic retention, low levels of nuclear p27, thus leading to decreased binding and inhibition of cyclin/CDK complexes and, eventually, increased cell cycle entry and proliferation [33, 34].

Altogether, these notions suggest two different hypotheses. One possibility is that loss of only one allele of CDKN1B in its C-terminal portion is sufficient to drive tumor progression, as already well established in mouse carcinogenesis models [29, 35]. Our recent data in mouse and human cancer cells support this hypothesis and suggest that p27 C-terminus, impacting on MT-dynamics via stathmin, can also regulate vesicle recycling, Ras signaling and, eventually, cell cycle entry and proliferation [17, 19, 33]. Similarly, the new work of Nguyen et al. support the evidence that p27-mediated MT-acetylation, via α-TAT1 regulation, is necessary for vesicle transport along axons, in neurons [12]. Moreover, it is possible that the lack of p27 C-terminus could lead to a decreased protein stability, as recently shown, for instance, in the case of T197A knock-in mice that display most of the phenotypes described in p27 null animals [36]. A second possibility is instead that the presence of at least one allele of CDKN1B is required for cancer progression, to guarantee the presence of a small amount of p27 in the cytoplasm. This hypothesis is supported by the characterization of a murine knock-in model, in which p27^KO^ was replaced by a p27^CK−^ allele, generating a p27^−/CK−^ mouse. These mice developed more hyperplastic lesions and tumors than their p27^WT^ and p27^KO^ counterparts [37]. Although lung and retina tumors, which mainly affected the p27^−/CK−^ murine model, are not related to the landscape of p27 mutations in humans [32], these results suggested that p27^CK−^ could act as an oncogene, independently of its function as a CKI [37].

A still unclear point in the biology of p27 is how (and why) a relatively short sequence of approx 100 amino acids can mediate so many interactions. Noteworthy, this sequence lacks clear functional domain(s), with the exception of the above-mentioned NLS. It has been reported that different regions of p27 protein retain the characteristics of intrinsically disordered regions (IDR), a peculiar non folded 3D conformation that confers the ability to bind to several different targets [38–40]. It is well recognized that the intrinsic lack of structure confers functional advantages, including the promiscuous interaction with a large number of “partners” and, also, the possibility to engage several upstream regulatory pathways leading to specific protein post-translational modifications. Noteworthy, p27 C-terminus contains several phosphorylable residues that are already known to determine its stability, localization and interaction preferences. It is therefore possible that the IDR conformation confers to p27 peculiar signaling and regulatory functions in different contexts, as the literature collected so far seems to indicate. The fact that many of these activities are related to the regulation of MT-dynamics supports the concept that p27 may act as a central relay, linking the control of cell division to other interphase MT-mediated processes, such as intracellular trafficking, cell motility, and differentiation.

## Conclusions

**Fig. 1:**
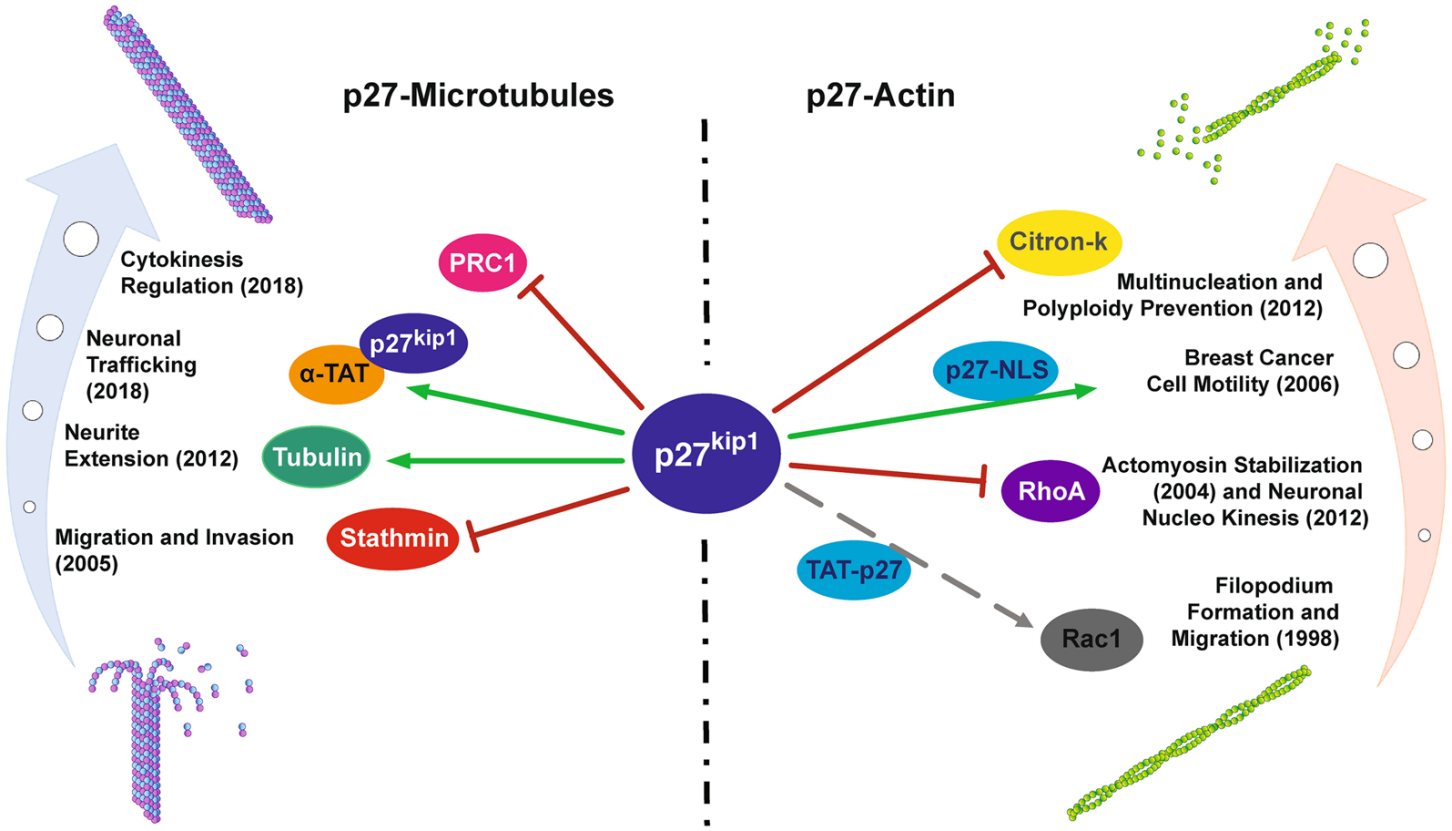
p27^Kip1^ regulates cytoskeleton dynamics. The main findings regarding the interaction of p27 with proteins that impact on cytoskeleton dynamics are depicted. On the left, p27^Kip1^ impacts on the microtubule cytoskeleton. p27 inhibits migration and invasion of neoplastic cells via stathmin [10], guarantees a proper extension of neurites [8], axonal transport velocity in neurons [12] and regulates cytokinesis limiting PRC1 activity [13]. On the right, p27^Kip1^ impacts on the actin cytoskeleton. The fusion protein TAT-p27 stimulates migration in hepatocellular carcinoma cells and correlates with Rac1 activity [5, 6]; p27 inhibits RhoA activity, determining actomyosin stabilization [7] and neuronal nucleokinesis [8]. p27-NLS variant, which accumulates into the cytoplasm, results in increased motility in MCF7 breast cancer cells [15]; p27 binds citron-kinase impinging on the downstream activation of RhoA, thereby regulating cytokinesis [9]

Recent evidences confirm that p27 exerts an important function on cytoskeleton, controlling actin and MT dynamics (Fig. 1). This function is carried out by binding and modulating the activity of many different interactors through its C-terminus, in both direct and indirect ways. Regarding the interaction with MTs, p27 is responsible for proper MT-acetylation, ensuring transport velocities in neuronal processes [12] and MT-bundling, required for physiological cytokinesis [13]. Moreover, p27 C-terminal truncated variants are not able to regulate MTs and lack the NLS, resulting in accelerated cell cycle progression. On the other hand, it is to note that p27 C-terminal truncated variants also lack the domains that regulate actomyosin polymerization, further impacting on p27 ability to control cytoskeleton dynamics. Therefore, the combined effects resulting from C-terminus mutation/truncation represent a strong oncogenic drive to determine increased motility, failure of cell-cycle inhibition and altered cell division. The reason why these mutations occur more frequently in specific types of cancer is something that remains to be fully understood and will be certainly at the center of future studies.

## Abbreviations

aa: amino acid(s)
α-TAT1: α-tubulin acetyl-transferase 1
CDK: cyclin-dependent kinase
CKI: cyclin-dependent kinase inhibitor
ECM: extracellular matrix
HDAC6: histone deacetylase 6
IDR: intrinsically disordered region
KO: knock-out
MEF: murine embryonic fibroblast
MT: microtubule(s)
NES: nuclear export signal
NLS: nuclear localization signal
PRC1: protein regulator of cytokinesis 1
p27^1–170^: p27 deletion mutant, lacking the C-terminus and the STM-binding domain
p27^1–190^: p27 deletion mutant, lacking the C-terminus and the PRC1-binding domain
p27^CK−^: p27 mutant, unable to bind cyclins and CDKs
ROCK: rho kinase
STM: stathmin
WT: wild-type
